# Magnetic-control multifunctional acoustic metasurface for reflected wave manipulation at deep subwavelength scale

**DOI:** 10.1038/s41598-017-09652-w

**Published:** 2017-08-22

**Authors:** Xing Chen, Peng Liu, Zewei Hou, Yongmao Pei

**Affiliations:** State Key Lab for Turbulence and Complex Systems, College of Engineering, Peking University, 100871 Beijing, China

## Abstract

Acoustic metasurfaces, exhibiting superior performance with subwavelength thickness, are ideal alternatives for functionalities such as wavefront modulation and acoustic energy trapping, etc. However, most of the reported acoustic metasurfaces were passive. Here a magnetically tuned mechanism is reported for membrane-type acoustic metamaterials. Harnessing the geometric nonlinearity of membrane structures, the transmission spectrum is both theoretically and experimentally tuned over broadband by an external static magnetic force. Simultaneously, the phase profiles can be readily tailored by the magnetic stimulus. Further, a magnetic-control multifunctional metasurface is proposed for low-frequency wave manipulation. By switching the magnetic force distribution, multi extraordinary phenomena, such as acoustic wave redirecting, focusing, bending, etc., are realized without changing the physical structure. Besides, it is demonstrated the proposed metasurface, at deep subwavelength scale (~1/85λ), supports anomalous reflected wave manipulation over a wide band. These results open up new degrees of freedom to steer acoustic wave and pave a way for designing active acoustic devices.

## Introduction

Recent years have witnessed the rapid development of artificially structured materials in which extraordinary phenomena are discovered^1–15^. In particular, acoustic metasurfaces have drawn significant attention due to the capabilities of phase engineering at will. Liang *et al*. proposed labyrinthine metasurfaces by coiling up space^16^. The incident wave was constrained to propagate along the curled channels, which resulted in arbitrary phase delay and a high refractive index. Resorted to these unique properties, fascinating acoustic wave manipulation, such as wave bending^17^, anomalous reflection and refraction^18–23^, converting radiation pattern^24^, etc., have been demonstrated both theoretically and experimentally. Inspired by these features, planar metasurfaces with subwavelength thickness are ideal candidates for constructing compact acoustic devices. However, most reported metasurfaces were based on the labyrinthine structures and operated in the frequency region above 2 kHz. This is due to that the thickness of labyrinthine metasurfaces is usually larger than 1/20 of the operating wavelength (>0.05λ). Thus, steering low-frequency acoustic wave (<1 kHz) calls for the emergence of deep-subwavelength acoustic devices. Besides, to realize desired phase shifts, the labyrinthine units for constructing acoustic metasurface need to be well-designed with different geometric sizes. The spatially varying configurations increase the complexity of manufacture and assembly.

Intellectualization is another developing trend of acoustic devices. However, most reported acoustic metasurfaces are passive and hampered by the lack of tuning capabilities. Another limitations of the subwavelength structures are the narrow operative band due to the high dispersion. To improve their performance, there exist a few preliminary studies on developing mechanisms for acoustic/elastic metamaterials with tunable effective parameters^25–32^. For example, Akl and Baz manufactured a fluid-solid composite structure containing piezoelectric active ingredients. As the voltage was applied to the piezoelectric elements, the effective dynamic density of this structure could be controllable^25^. Other approaches involve the exploitation of mechanical deformation for specific configurations, resulting in the tunable band structure for elastic wave^31^. Nonetheless, a robust method for realizing wide tuning range is still lack, especially for airborne sound. Driven by the development of tunable metamaterials, the research agenda is shifting towards designing acoustic devices with tunable and switchable functionalities. Recently, Popa *et al*. demonstrated that the transmitted acoustic field patterns, through a metamaterial slab, can be entirely changed in real time by configuring the digital electronics^33^. However, the device suffered from limited tuning range due to the bandwidth of the bandpass filter. Thus, other type of acoustic devices are desirable for broadband tunability.

In the following, we design a magneto-mechanical tunable metamaterial which is composed of an elastic membrane and a soft magnetic central mass. When a static magnetic load is applied, the tuning ratio of the measured transmission spectrum and phase is about 200%. The mechanism can be illustrated by a static analysis, and it is found that the improvement of structural stiffness plays an important role. Finally, an acoustic metasurface, constructed by the tunable metamaterial with a rigid back cavity, is used to steer the reflected acoustic wave in the low frequency. Anomalous reflected wave manipulation is numerically demonstrated over a wide band. Besides, abundant wave propagation modes can be switched by tuning the magnetic force distribution. The results show the versatility of the tunable acoustic metasurfaces and have potential in acoustic imaging, cloaking and sensing applications.

## Results

### Tunable mechanism for membrane-type acoustic metamaterials

**Figure 1:**
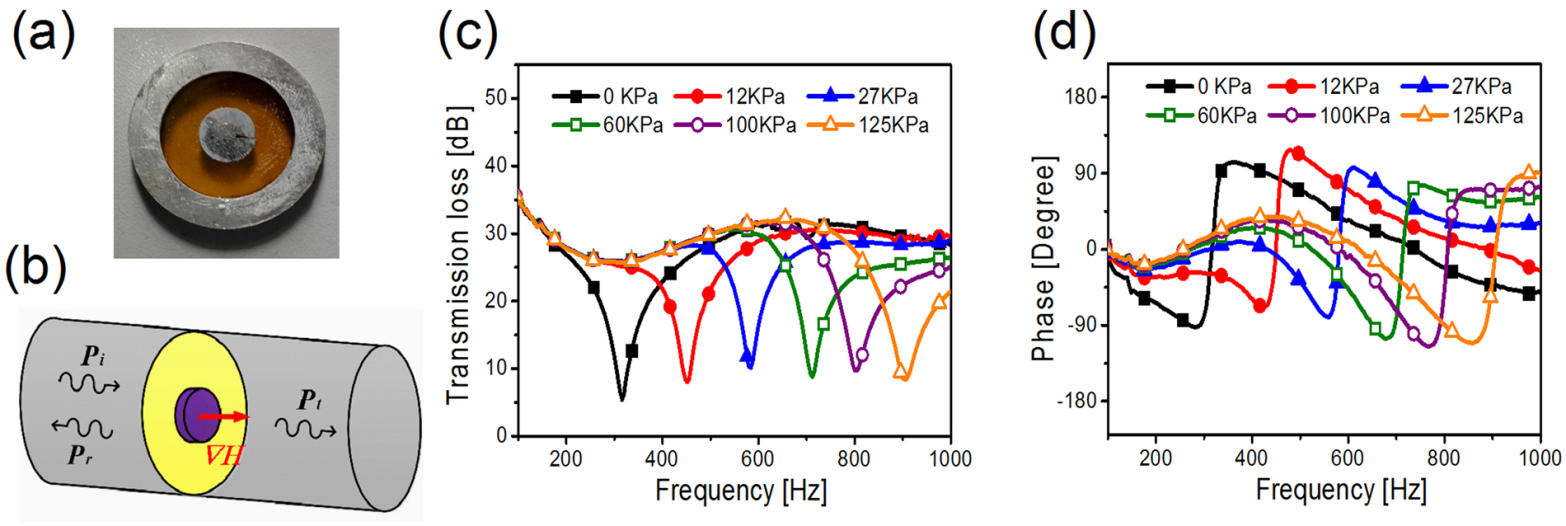
(**a**) Photo of the mass-weighted membrane structure. (**b**) The schematic of the experimental measurement device. (**c**) The measured acoustic transmission loss of the mass-weighted membrane structure under different magnetic forces. (**d**) The measured phase of the mass-weighted membrane structure under different magnetic forces.

For airborne sound, the configurations of acoustic metamaterials (AMs) can roughly divided into three types, i.e., membrane-type AMs^34–37^, Helmholtz resonators^38, 39^, and space-coiling AMs^40^. However, latter two types are fabricated with hard materials whose properties cannot be easily adjusted. For this sake, membrane-type AMs are selected as the carriers for realizing tunable properties in this paper. The mass-weighted membrane structure, fixed by a support frame, is composed of a central mass and an elastic membrane as shown in Fig. 1(a). The elastic membrane, with a radius *a* of 10 mm and thickness *t* of 0.1 mm, is made of polyamide. Young’s modulus *E*, density ρ and Poisson’s ratio *v* for the membrane material is 20 MPa, 980 kg/m^3^ and 0.49, respectively. The central mass, with a 6 mm diameter, is specially selected as an iron disk of 310 mg. The support frame, made by a pair of circular aluminum rings with an outer diameter of 29 mm, is designed to fit snugly in the testing apparatus.

Considering magnetic control strategies have the advantages of non-contact and low voltage, we devote to developing a magnetic tunable method for acoustic metasurfaces. The acoustical property of the mass-weighted membrane structure is experimentally investigated in a static magnetic field as shown in Fig. 1(b). By adjusting the relative distance between the magnet and the sample, different out-of-plane magnetic forces are applied on the central mass. As shown in Fig. 1(c), the transmission loss spectra are highly dispersive and tuned over a wide range (~200%). The transmission loss dip, corresponding to the resonant frequency, shifts from 316 Hz to 904 Hz with the increase of magnetic force. What’s more, the phase can be tailored simultaneously by magnetic field as shown in Fig. 1(d). Below the resonant frequency, the negative acceleration of the acoustic surface leads to the negative phase. When the excitation frequency sweeps through the resonant frequency, a sharp jump occurs due to the reverse of oscillation.

**Figure 2:**
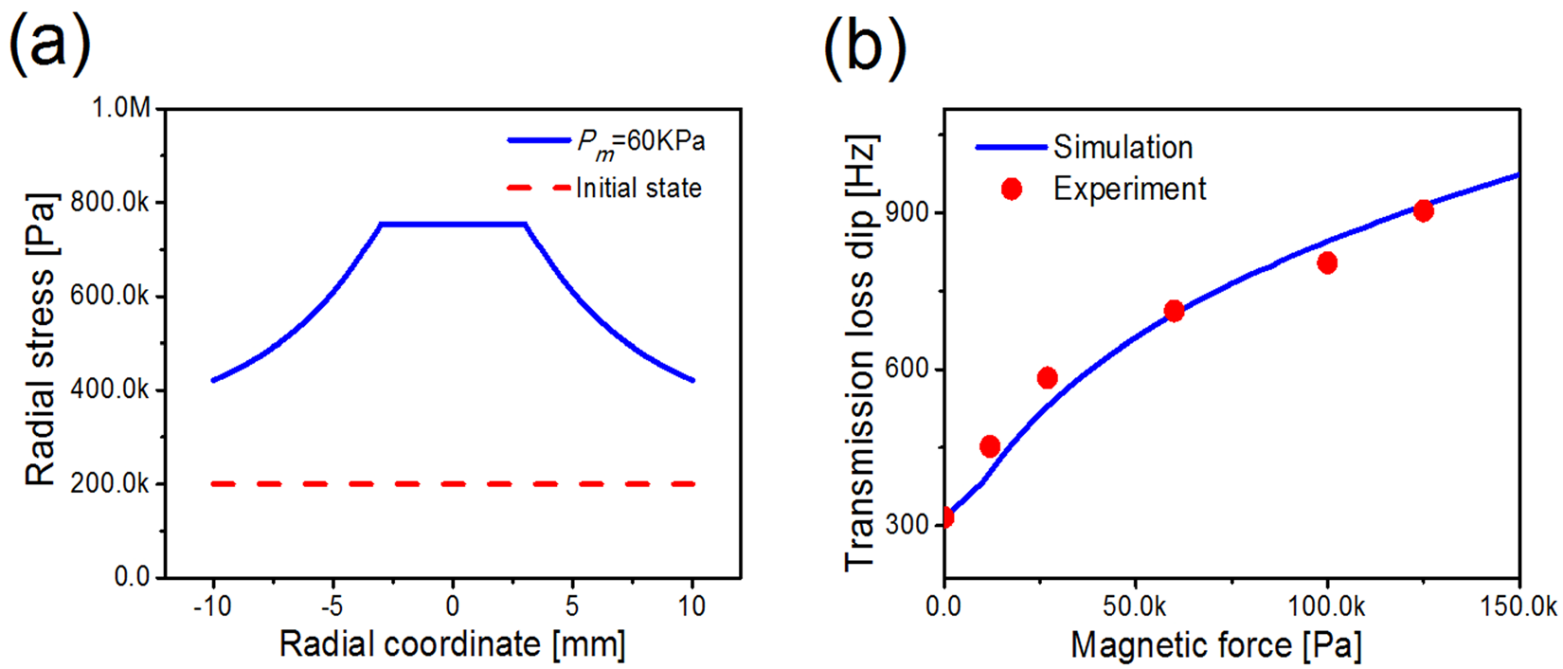
(**a**) The numerical results of radial stress distribution with/without out-of-plane magnetic force. The initial state is uniform stress as shown in red dash curve, and blue solid curve denotes simulation result of *Pm* = 60 KPa. (**b**) The influence of out-of-plane magnetic force on the transmission loss dip. The experimental results are shown in red circles, and blue solid curve denotes simulation result.

To illustrate the mechanism of the tunable phenomenon, a preliminary static analysis is conducted. In this case, we suppose that the elastic membrane, subjected to initial uniform tension *σ*_0_, is perfectly bonded with the mass. Due to extreme modulus difference, the mass acts as a rigid body. When a static pressure ${P}_{m}={\mu }_{0}M\nabla H$ is applied on the mass, the membrane experiences large deflection and small bending angle. It needs to be stressed that the strong geometric nonlinearity is a unique properties for membrane-type structures, which distinguish from other structural forms. Thus, the strain can be expressed in polar coordinate^41^:

1 $${\varepsilon }_{r}=\frac{du}{dr}+\frac{1}{2}{(\frac{dw}{dr})}^{2}\,{\rm{and}}\,{\varepsilon }_{\theta }=\frac{u}{r}$$

where *u* and *w* are the radial and out-of-plane displacements of the membrane, respectively. It reveals that the nonlinearity term is mainly determined by the out-of-plane displacement. Due to the axial symmetry, the balance equation in circumferential direction is automatically satisfied. Meanwhile, the relation between the bending angle and the deflection is introduced by $\sin \,\theta =-\frac{dw}{dr}$. The equilibrium equations are:

2 $$2\pi r{\sigma }_{r}t\frac{dw}{dr}=-\pi {R}_{1}^{2}{P}_{m}\,{\rm{and}}\,\frac{d}{dr}(r{\sigma }_{r})-{\sigma }_{\theta }=0$$

where *σ*_*r*_ and *σ*_*θ*_ are the radial and circumferential stresses of the membrane, respectively. The stress-strain relationship is captured by linear constitutive equations:

3 $${\sigma }_{r}={\sigma }_{0}+{\sigma }_{1}(H)=\frac{E}{1-{\nu }^{2}}({\varepsilon }_{r}+\nu {\varepsilon }_{\theta })\,{\rm{and}}\,{\sigma }_{\theta }={\sigma }_{0}+{\sigma }_{2}(H)=\frac{E}{1-{\nu }^{2}}({\varepsilon }_{\theta }+\nu {\varepsilon }_{r})$$

where *σ*_1_ and *σ*_2_ are the additional stresses induced by out-of-plane loads. To solve the nonlinear problem, the numerical method is implemented. Firstly, the radial stress *σ*_*r*_ along the membrane is calculated in Fig. 2(a). When a magnetic force of 60 kPa is generated, the membrane becomes taut and the tension nonuniformly improves. Since the largest rotating angle occurs in the edge of the mass, there exists a maximum of the tension. It is noted that the magnetic force can significantly alter the stress condition of membrane, resulting in broadband tunability. For membrane-type structures, tension plays an important role in their acoustical properties. Thus, the influence of magnetic force on the transmission loss dip is further explored as shown in Fig. 2(b). It is observed that the experimental results agree well with the simulations. As the magnetic force increases, the transmission loss dip varies with the improved structural stiffness. In a word, broadband tunable method is proposed by harnessing the magnetic-induced geometric nonlinearity.

### Design of the unit cell for reflected wave

**Figure 3:**
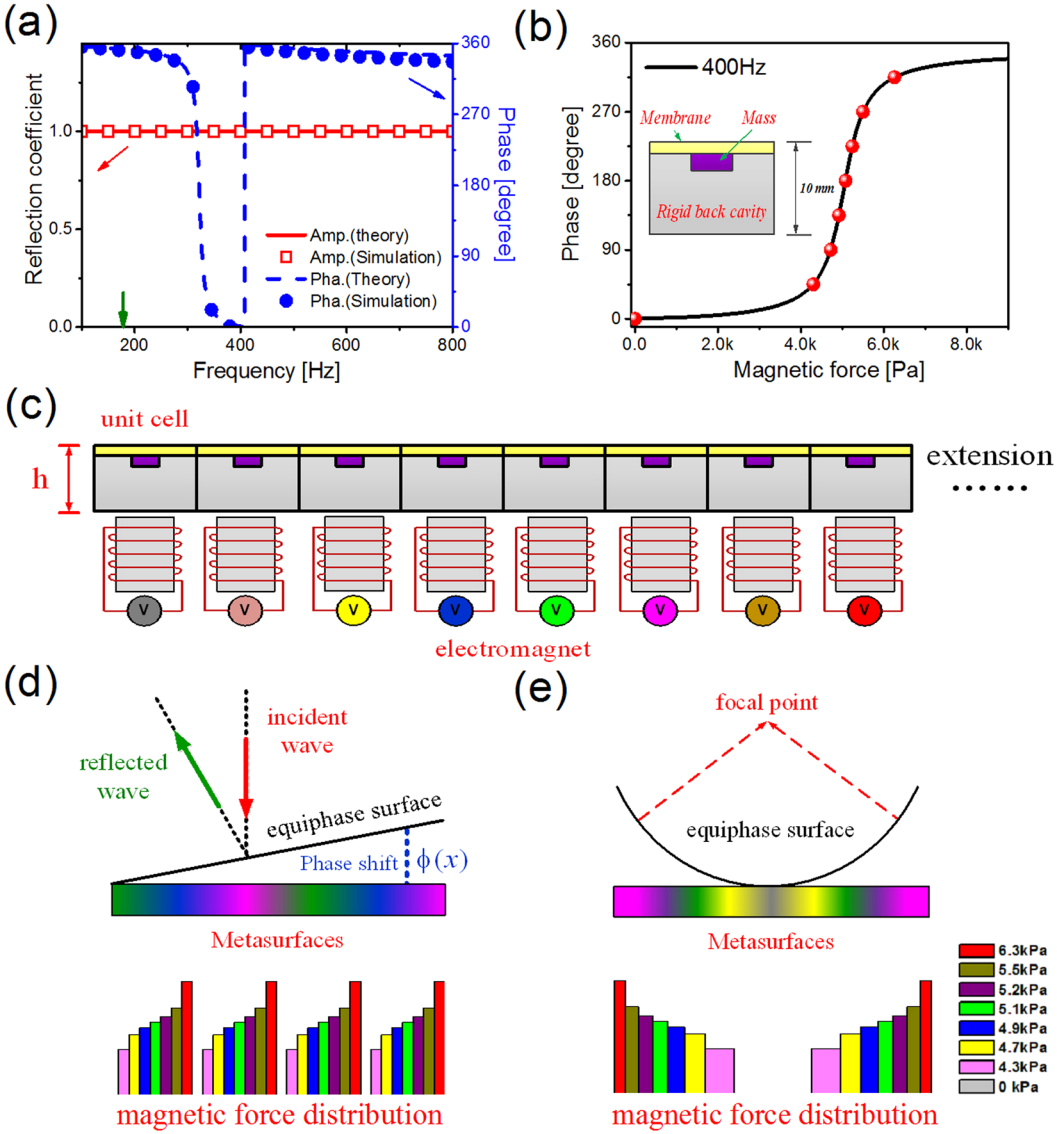
(**a**) Theoretical and numerical solutions of the reflection coefficient and phase. The green arrow indicates the original eigenfrequency of the membrane structure. (**b**) The effect of the magnetic force on the reflected wave phase in 400 Hz. The red circles represent eight control modes, in which discrete phase shifts cover the full 2π span with steps of π/4. Inset: the schematic diagram of the unit. (**c**) The schematic diagram of the proposed magnetic control metasurface. The different colors represent varied input voltages, which results in different magnetic forces. The schematic diagram of (**d**) anomalous reflection and (**e**) focusing are plotted. The bar graphs show the desired magnetic force distributions for achieving related functionalities in 400 Hz.

Two key factors for wave manipulation are phase modulation and transmission efficiency. However, most resonant metamaterials suffer from high dispersion. To enhance the energy of reflected wave, a rigid back cavity is introduced as shown in the inset of Fig. 3(b). The back cavity, with a depth *h* of 10 mm, is sealed by the tunable membrane-type structure. The main idea is that the phase delay is tuned by coupling the incident wave with the membrane, while the rigid wall is used to realize total reflection. To verify the concept, the reflective characteristic of the unit cell is understood by the acoustic impedance method^8^. Considering that only the piston-like perturbation of the structure can propagate to far field, the surface-averaged Green function $\langle {G}_{m}\rangle$ is concerned. Using the modal superposition method, $\langle {G}_{m}\rangle$ can be expressed as^42, 43^:

4 $$\langle {G}_{m}\rangle =\frac{\langle W\rangle }{\langle \delta p\rangle }=\sum _{i=1}\frac{S < {W}_{i}(r,H){ > }^{2}}{\iint \rho {W}_{i}^{2}dS\cdot [{\omega }_{i}^{2}(H)-{\omega }^{2}]}$$

where $\langle W\rangle$ is the surface-averaged displacement of the membrane-type structure, *δ*_*p*_ denotes the sound pressure variation through the structure, *S* refers to the relevant area, ρ is the local surface density, *W*_*i*_ is the *ith* eigenmodes, and *ω*_*i*_ is relevant angular eigenfrequency. When the magnetic field is imposed, both the eigenmodes and the eigenfrequencies are varied. According to the definition, the impedance of the membrane-type structure is given by ${Z}_{m}={(-i\omega \langle {G}_{m}\rangle )}^{-1}$. Due to the unit cell is orders of magnitude smaller than the relevant wavelength, the air in the back cavity is undergoing uniform compression and expansion in response to the membrane’s movement. Similarly, the impedance of the back cavity can expressed as ${Z}_{c}={(-i\omega h/{Z}_{0}{c}_{0})}^{-1}$, with *Z*_*o*_ denoting air acoustic impedance and *c*_*o*_ denoting sound velocity. Thus, the total impedance of this series system is *Z*_*h*_ = *Z*_*m*_ + *Z*_*c*_, and the reflection coefficient is calculated by:

5 $$R=\frac{{Z}_{0}-{Z}_{h}(H)}{{Z}_{0}+{Z}_{h}(H)}$$

The reflection coefficient and phase are present in Fig. 3(a), where the theoretical solutions can exactly consist with the numerical solutions. Ignoring the effect of the dissipative losses, the energy can be effectively reflected in the whole frequency band due to the impedance mismatch. Particularly, the resonant frequency shifts to higher level under the effect of the back cavity, which is a hybrid resonance phenomenon^8^. Below the hybrid resonant frequency, the membrane structure’s vibration is in accord with the incident wave and the reflected phase is lagged (180 < ϕ < 360). Above the hybrid resonant frequency, the membrane structure’s vibration is opposite with the incident wave and the reflected phase advance is obtained (0 < ϕ < 180). Thus, Inferred from Eq. (5), desired phase delay can be controlled by tuning the magnetic field. Without configuration transformation, the structural stiffness is easily tuned by the proposed tunable mechanism.

To efficiently regulate the reflected acoustic wave, the unit cell should satisfy the conditions of tunable phase delay over the whole 2π range. To show the capability of low-frequency wave control, we begin with the frequency of 400 Hz. From Fig. 3(b), it demonstrates that the magnetic force can readily tailor the phase profiles. When the resonant frequency of the unit cell is well above or well below 400 Hz, the membrane keeps nearly motionless and reflects the wave without phase aberration. As the resonant frequency closes to 400 Hz, the reflected wave is strongly coupled with the membrane and high phase delay is observed. The phase advance is obtained for small magnetic force at 400 Hz, when the resonant frequency of the structure is below 400 Hz. Increasing the magnetic force, the structural stiffness is improved and the resonant frequency is above 400 Hz. The desired phase delay is realized at 400 Hz. What’s more, the magnetic force is sufficiently small which means low energy input.

To replace the continuous phase profiles in theory, the eight control modes, possessing discrete phase shifts with steps of π/4, is utilized for reflected wave manipulation in 400 Hz. Subsequently, the eight control modes are represented by different magnetic forces as shown in Fig. 3(b). When the applied magnetic field is appropriate, discrete control modes can be obtained in other frequency range. More details about controlling the reflected phase of the unit are discussed in the Supplementary Note 1.

### Broadband versatility of reflected wave manipulation at deep wavelength scale

**Figure 4:**
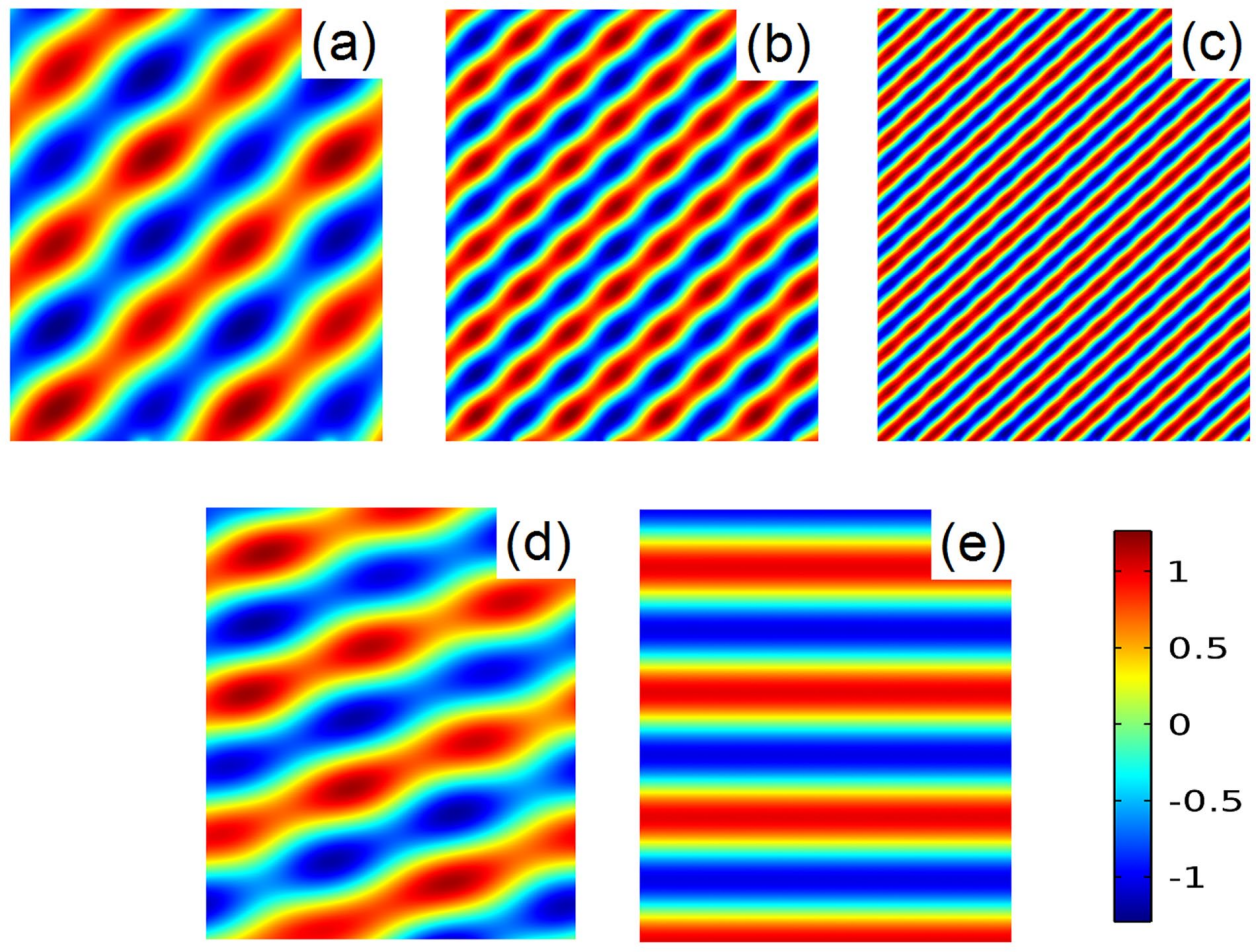
For the design reflected angle with 42 degree, reflected pressure field patterns are plotted in the frequency of (**a**) 400 Hz, (**b**) 800 Hz and (**c**) 1600Hz. By magnetically tuning the phase profiles, the reflected angles of (**d**) 19 degree and (**e**) 0 degree are realized in 400 Hz.

The proposed acoustic metasurface is constructed with 256 unit cells as shown in Fig. 3(c). These unit cells, with the period constant *p* of 20 mm, are arranged in x direction and controlled independently by the electromagnets. The wavelength λ_0_ in 400 Hz is defined as reference wavelength. The thickness of the slab is 10 mm (~1/85λ_0_), which is at deep wavelength scale. Compared with common metasurfaces realizing phase shifts by adjusting the geometric size of each unit, the phase delays are tuned by stress states for the proposed metasurface. Firstly, the broadband property of the proposed planar metasurface is investigated through wave redirecting. As shown in Fig. 3(d), it is supposed that a linear phase shift exists along the metasurface. Due to the introduction of phase discontinuity at the interface, it allows us to revisit the law of reflection by applying Fermat’s principle. For simplicity, an incident plane wave, propagating along the negative y direction, is investigated. This condition will be satisfied in the following calculations, unless otherwise specified. As there exists gradient phase shifts along the x direction, the angle of the reflected wave *θ*_*r*_ (measured from the y direction) can be deduced^44^:

6 $${\theta }_{r}=arc\,\sin (\frac{1}{k}\frac{d\varphi (x,H)}{dx})$$

where ϕ(*x,H*) represent the phase shift, and $k=2\pi /\lambda$ is the wave vector in air. It is noted that the equiphase surface is equivalent to the case of an inclined plane. Thus, the anomalous reflected wave is achieved and breaks traditional Snell’s law. To demonstrate this feature, spatially periodic magnetic force distribution is implemented as shown in Fig. 3(d). When the desired reflected angle is 42 degree, the gradient of phase should be equal to π/32*p* in 400 Hz. When the gradient of phase is chosen as dϕ/d*x* = π/32*p*, it indicates that there are eight identical units for each discrete phase shift of π/4. To obtain the constant reflected angle, the metasurface should be adapt to the different phase profiles. Guided by the Eq. (6), desired phase gradients (π/16*p* for 800 Hz and π/8*p* for 1600Hz) are achieved by tuning the magnetic field. The reflected pressure field patterns are numerically investigated in Fig. 4(a),(b) and (c). The calculated reflected angle shows excellent agreement with the theoretical value, which can also refer to the Supplementary Note 2. The broadband availability is demonstrated for reflected direction control. What’s more, the reflected direction can be easily tuned by the magnetic field. To shift the wave toward the normal direction in 400 Hz, the phase gradients are adjusted with the value of $\pi /64p$ and 0, respectively. The relevant reflected angels are observed as 19 degree and 0 degree in Fig. 4(d) and (e). As the gradient of phase decreases, the slope of the equiphase surface slows down. Thus, the angle of the reflected wave gradually gets close to the normal direction of the slab. Furthermore, the functionality of sound field scanning by static magnetic field is present in the Supplementary Movie 1.

Meanwhile, the convergence of the reflected wave is also a fascinating phenomenon, which has potential application for acoustic imaging and nondestructive testing. It will be proved that the proposed metasurface can simultaneously be used for planar focusing. To achieve acoustic focusing by a planar structure, the arc-shaped phase profile is desired on the metasurface. For a given focal length *f*, the phase distribution can be determined by the geometrical relation:

7 $$\varphi (x)=k(\sqrt{{x}^{2}+{f}^{2}}-f)$$

**Figure 5:**
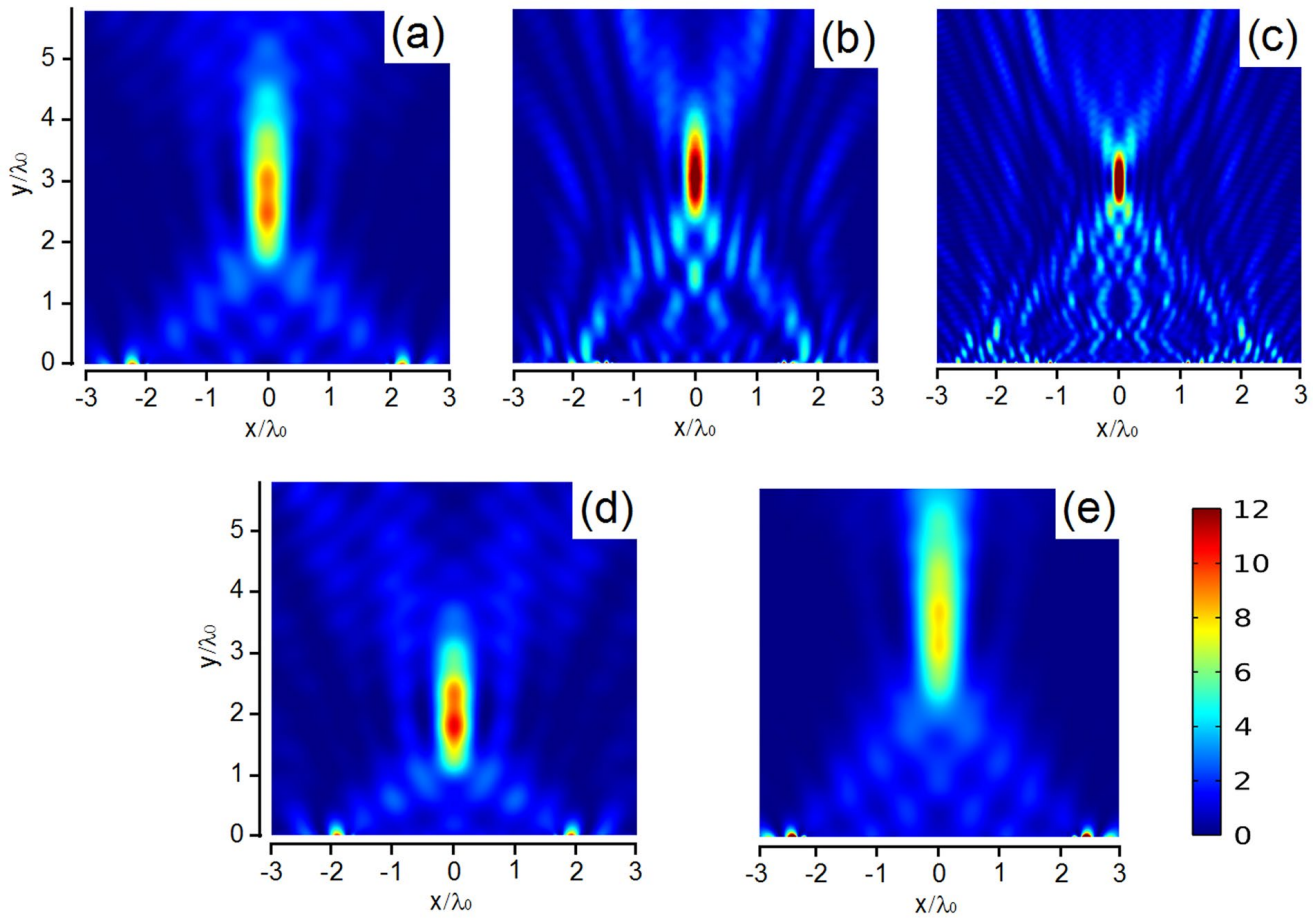
For the design focal length with 3λ_0_, the reflected pressure intensity in the frequency of (**a**) 400 Hz, (**b**) 800 Hz and (**c**) 1600Hz. By changing the magnetic field distribution, the focal lengths are located near (**d**) (0, 2λ_0_) and (**e**) (0, 4λ_0_) in 400 Hz.

When the unit cell is far from the acoustic axis, phase advance is required for compensating acoustic path difference. For the situation of a focal spot located at (0, 3λ_0_) in 400 Hz, desired reflected wavefront is calculated from Eq. (7). Eight discrete modes, mentioned above, are adopted to imitate the continuous phase profile. Accordingly, the magnetic forces with symmetric distribution are applied as shown in Fig. 3(e). To examine the effectivity in broadband, the magnetic fields are elaborately designed for 800 Hz and 1600 Hz, respectively. The reflected pressure field pressure intensity are plotted in Fig. 5(a),(b) and (c), and the focusing spots are clearly observed. The slight deviation between calculated focal length and theoretical value is observed due to the discrete phase profile. Similarly, the focal spot can be regulated based on the demand. To realize a focal spot located at (0, 2λ_0_) or (0, 4λ_0_), the radius of the equiphase surface is changed by adjusting the magnetic force distribution. More details about the magnetic field design are listed in the Supplementary Note 3. The acoustic energy is concentrated as shown in Fig. 5(d) and (e). The capability of focal spot control at will is also demonstrated in the Supplementary Movie 2.

**Figure 6:**
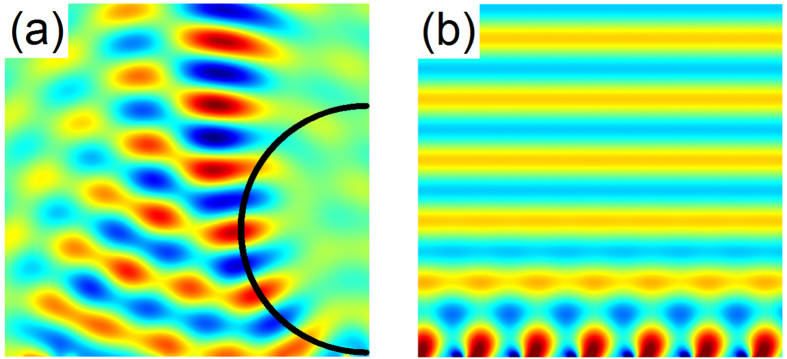
Reflected pressure field patterns in 400 Hz. (**a**) Acoustic wave bending along a half-circle trajectory and the design radius is 2λ_0_. (**b**) Conversation of a propagating wave into a surface wave.

Stemming from the new degree of freedom by the magnetic field, the versatile wavefront manipulation is achieved without changing the physical structure. Here, the multifunction of the metasurface is extended to acoustic wave bending and propagation mode conversion. The formation of the bending beam can be understood as a family of delicately arranged rays concentrating on a curved trajectory. To guide sound energy along curved paths, the desired trajectory is retrieved from tracing each individual caustic ray. The condition for generating a bending acoustic beam is predicted by the caustic theory. For a half-circle trajectory, the phase profile follows the relation: $\varphi (x)={k}_{0}(x-2r\sqrt{x/r})$. To simulate the continuous phase distribution, the magnetic forces are properly adjusted (see in the Supplementary Note 4). As shown in Fig. 6(a), the reflected pressure beam occurs bending. Finally, the phenomenon of conversing a propagating wave into a surface wave is explored. It is noted that the wave vector *k*_*x*_ along the x direction increases with the phase gradient. For the condition *k*_*x*_ > *k*_0_, the acoustic wave decays along the normal direction and is confined to propagate along the surface. Thus, the phase gradient of π/20*p* is employed by the magnetic field. It is predicted by Eq. (6) that the reflected angle is 90 degree. In Fig. 6(b), the formation of the surface wave propagating is clearly observed. In this paper, we have not been focused on the dissipation effect. However, the phenomena of wavefront manipulation can also be realized with the existence of the dissipation. More details about the influence of the dissipation effect on steering wavefront are discussed in the Supplementary Note 5.

## Discussion

In summary, a membrane-type acoustic metasurface with the deep-subwavelength thickness (~1/85λ_0_) is proposed, which is vital to steer low-frequency sound wave. To overcome the limitation of narrow operative band, a magnetic tunable mechanism is developed for the metasurface. The geometric nonlinearity of membrane structures is intentionally exploited, which results in a wide tuning range. The robust effect is verified both experimentally and theoretically. By tailoring the phase profiles with the magnetic field, the acoustic wave redirecting and focusing are demonstrated over a broadband. Beyond that, the proposed acoustic metasurface is highlighted for the versatility of wavefront modulation in real time. The complex propagation modes, such as wave bending and excitation of surface wave, can be readily switched by tuning the magnetic forces. The design concept achieves wave manipulation with new degree of freedom, and may provide an enlightening insight for the new generation of programmable devices.

## Methods

### Experiments

To apply a gradient magnetic field to the sample, a hollow cylindrical magnet spliced by 14 small magnets (NdFeB) was elaborately fabricated. The magnetic field was varied along the axial direction and the maximum magnetic force occurred at the end face of the magnet. The detail of the magnetic field distribution along the axial direction can be found in our early work^27^. Under the non-uniform magnetic field, the magnetic force per unit volume can be calculated by *P*_*m*_ = *μ*_0_ *M*∇*H* ^45^. It is noted that both the magnetization state *M* and magnetic field gradient ∇*H* affect the magnitude of the magnetic force. Thus, the magnetic force can be tuned by varying the relative location between the magnet and the sample. The acoustic transmission spectrum and phase of the sample were measured by the commercial instrument (Brüel & Kjær type-4206-T). Considering the magnetic field may cause adverse effect on sound pressure measurements, the microphones were located away from the magnet by elongating the impedance tube with an additional aluminum tube. Systematic errors in the measurements mainly arise from the sound leakage due to the assembly process.

### Numerical simulations

The simulations were performed with the commercial finite element analysis solver COMSOL Multiphysics. For unit cell calculations, a fixed constraint boundary condition was set for the rim of membrane, while the pre-stretching of the membrane was realized by a 0.2 MPa initial stress under geometric nonlinearity. The initial stress was determined by the experimental results in Fig. 1(c). In addition, the magnetic force was reduced to boundary load which was applied on the surface of the central mass. Plane wave radiation boundary condition was applied on the incident boundaries and the periodic boundary condition was employed in the y direction to calculate the pressure field distribution (Fig. 4). For acoustic focusing, wave bending and mode conversion simulations (Figs 5, 6), perfectly matched layers (PMLs) and background pressure field were utilized to eliminate the reflected waves by the outer boundaries because of the plane wave radiation boundaries were not sufficient to absorb the waves with oblique incidence.

## Electronic supplementary material

Supplementary information

The functionality of sound field scanning is achieved by the static magnetic field

The capability of focal spot control at will
