# Supplementary material for: Magnetic-control multifunctional acoustic metasurface for reflected wave manipulation at deep subwavelength scale
Source: Sci Rep. 2017 Aug 22;7:9050. doi: 10.1038/s41598-017-09652-w (PMC5567380; doi:10.1038/s41598-017-09652-w)
Supplement: Supplementary file 1 — Supplementary information [file 41598_2017_9652_MOESM1_ESM.docx]

**Supplementary material of “Magnetic-control multifunctional acoustic metasurface for reflected wave manipulation at deep subwavelength scale”**

Xing Chen^1^, Peng Liu^1^, Zewei Hou^1^, Yongmao Pei^1^

*^1^State Key Lab for Turbulence and Complex Systems, College of Engineering, Peking University, Beijing 100871, China*

**SUPPLEMENTARY FIGURES**


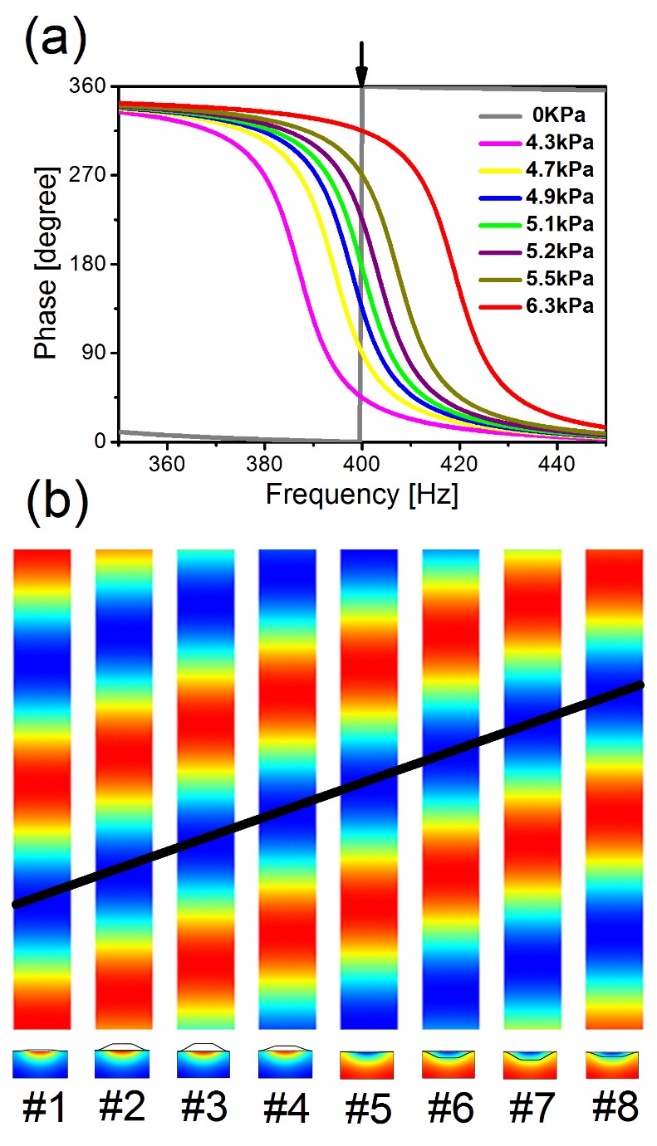


**Supplementary Figure 1 The reflected properties of the eight discrete units in 400Hz.** (a) The phase of the unit with different magnetic loads is plotted. (b) The reflected acoustic pressure patterns of the eight discrete units in 400Hz. In the design frequency (i.e. 400Hz), the reflected phase can span the 2π range with the increment of π/4. For different units, the structural vibration and the total acoustic pressure are given in the bottom. The black line is used to represent the linear variation of the phase.


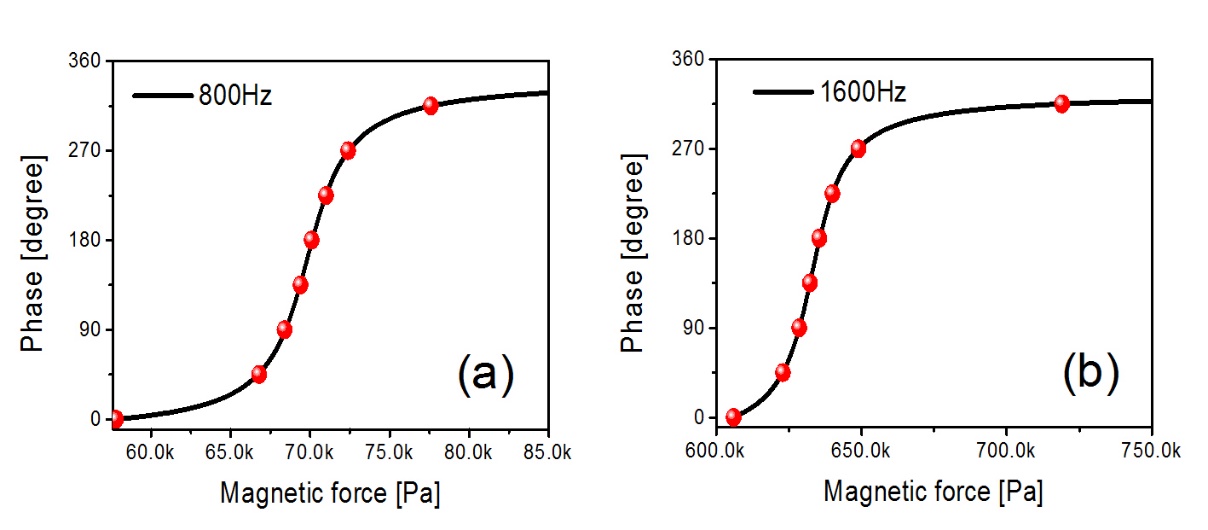


**Supplementary Figure 2 Magnetic-control phase delay in a wide band.** The effect of the magnetic force on the unit’s reflected wave phase in (a) 800Hz and (b) 1600Hz. The red circles represent eight states, in which discrete phase shifts cover the full 2π span with steps of π/4.

**
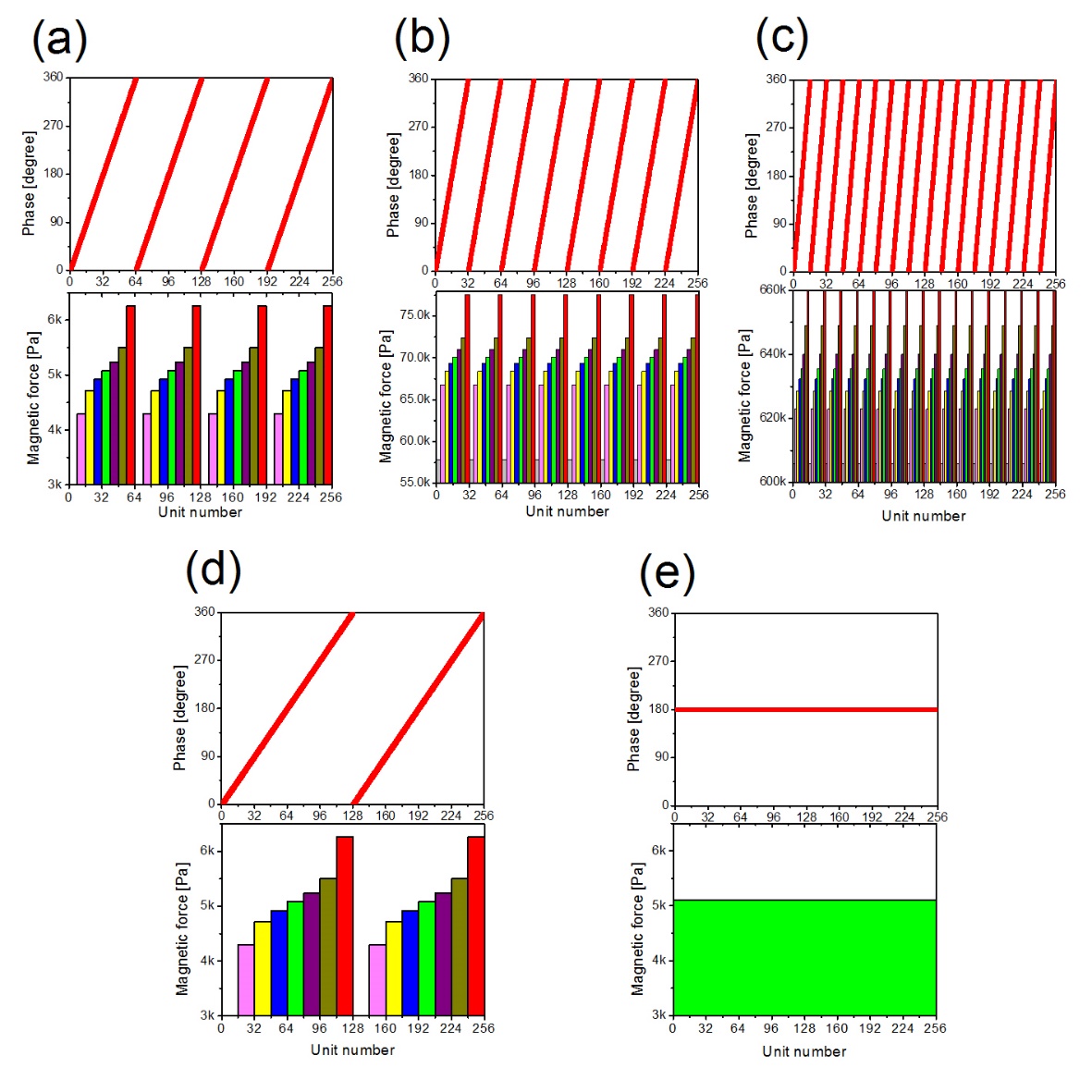
**

**Supplementary Figure 3 Magnetic-control phase profiles for anomalous reflection.** For the design reflected angle of 42 degree in (a) 400Hz, (b) 800Hz and (c) 1600Hz, both the theoretical phase profiles and the applied magnetic forces are given. To realize the reflected angles of (d) 19 degree and (e) 0 degree in 400Hz, magnetically tuning the phase profiles are plotted.

**
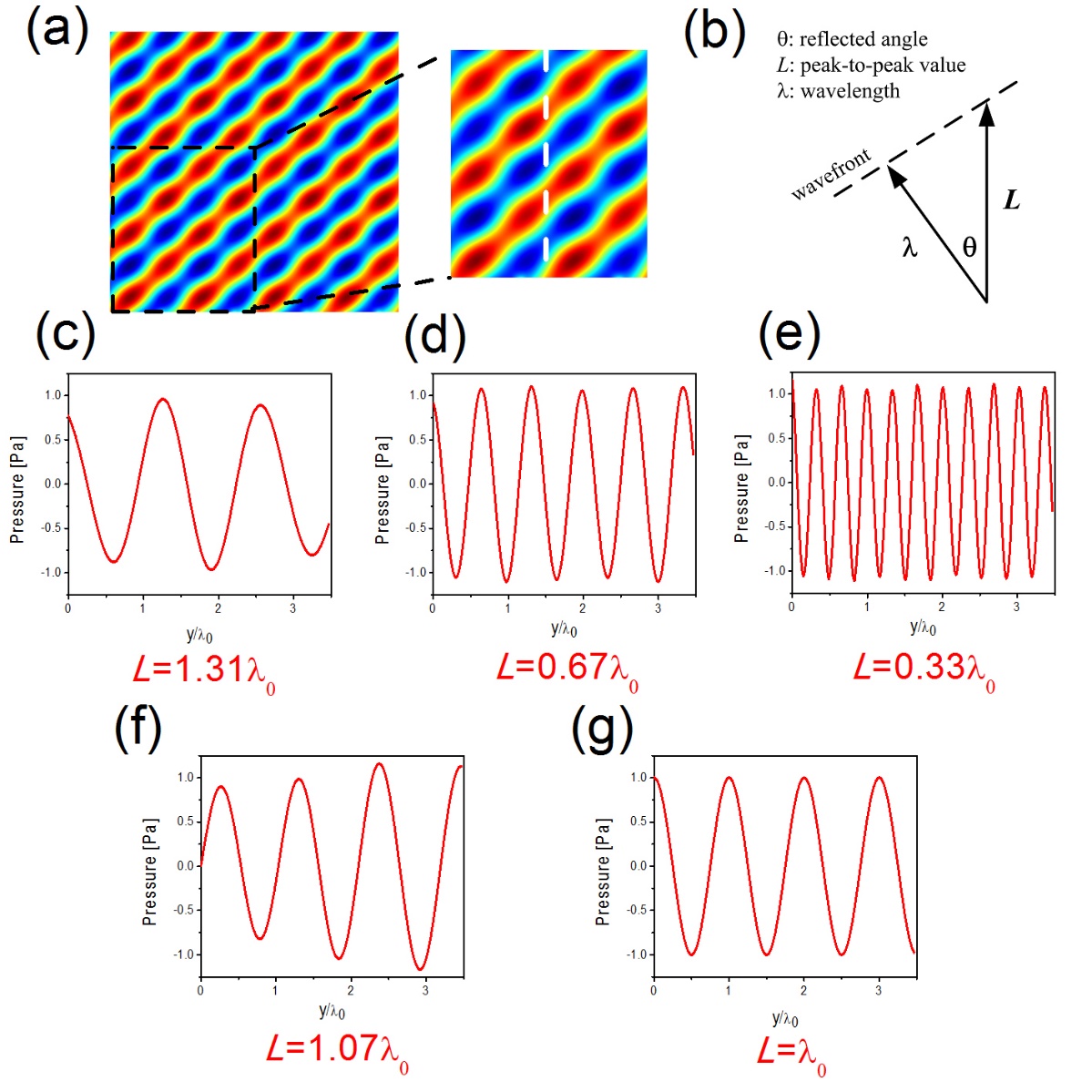
**

**Supplementary Figure 4 Calculation of the reflected angle.** (a) To show the patterns of anomalous reflection clearly in high frequency, partial region (the dotted box) is given in the manuscript. (b) The geometrical relationship between the reflected angle and the peak-to-peak distance along the y direction. (c)-(g) The pressure distributions (along the white dotted line) for different magnetic-control reflected field patterns are plotted, which correspond to the Fig. 4 in the manuscript.

**
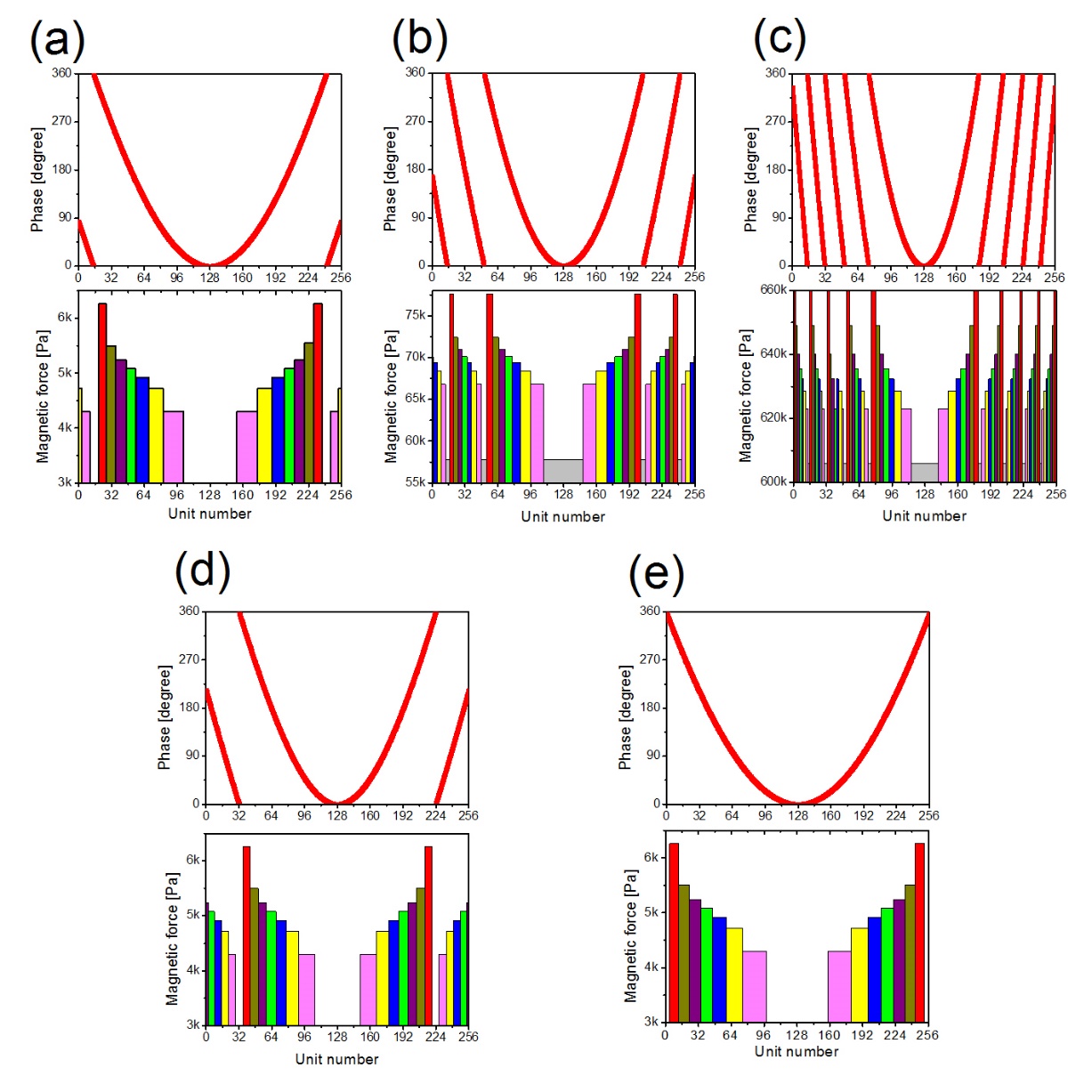
**

**Supplementary Figure 5 Magnetic-control phase profiles for planar focusing.** For the design focal spot located at (0, 3λ_0_) in (a) 400Hz, (b) 800Hz and (c) 1600Hz, both the theoretical phase profiles and the applied magnetic forces are given. To realize tunable focal lengths of (d) 2λ_0_ and (e) 4λ_0_ in 400Hz, the magnetic-control phase profiles are plotted.

**
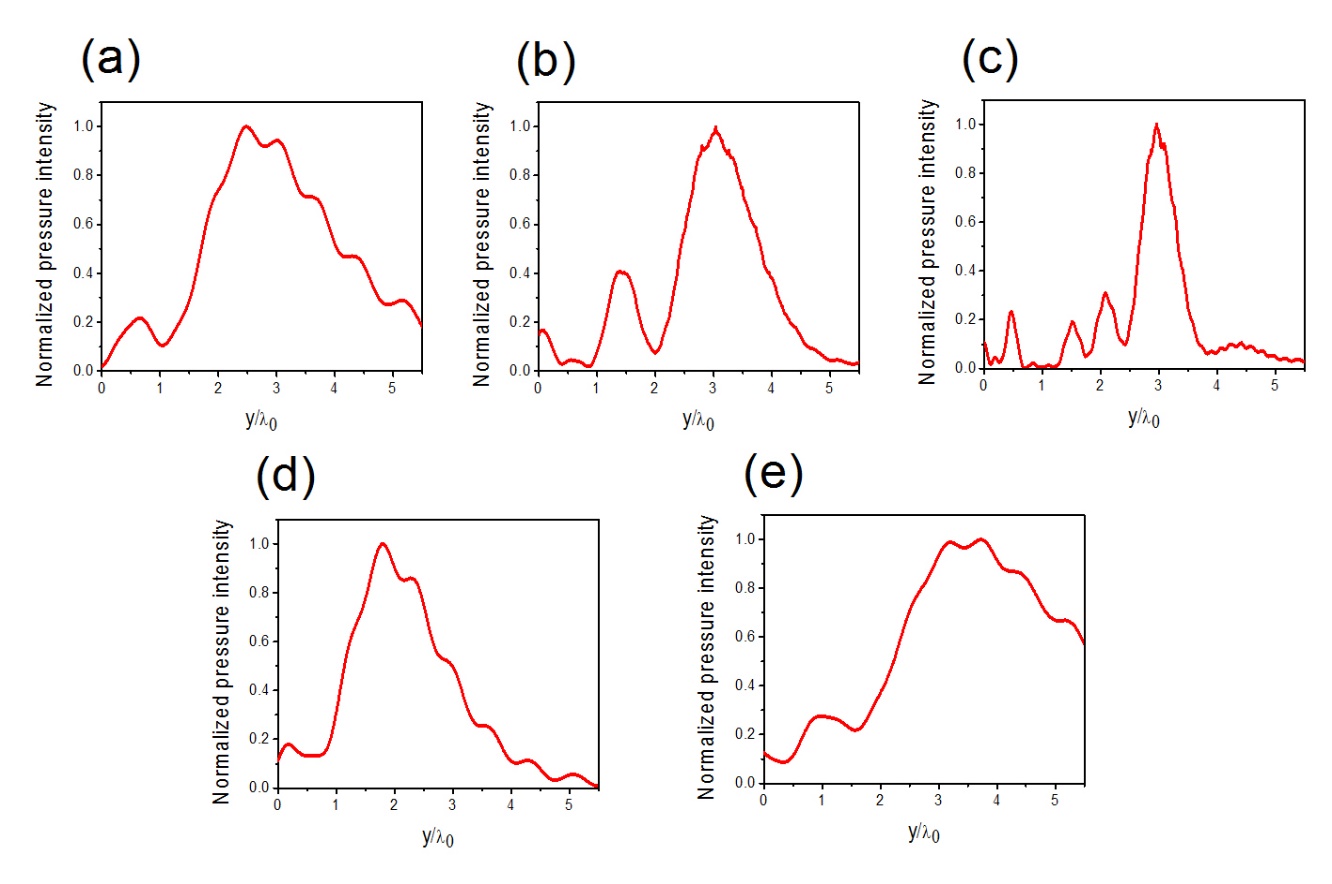
**

**Supplementary Figure 6 The pressure intensity along the acoustic axis** (a)-(e) The pressure intensity (along x=0) for different magnetic-control reflected patterns are plotted, which correspond to the Fig. 5 in the manuscript.

**
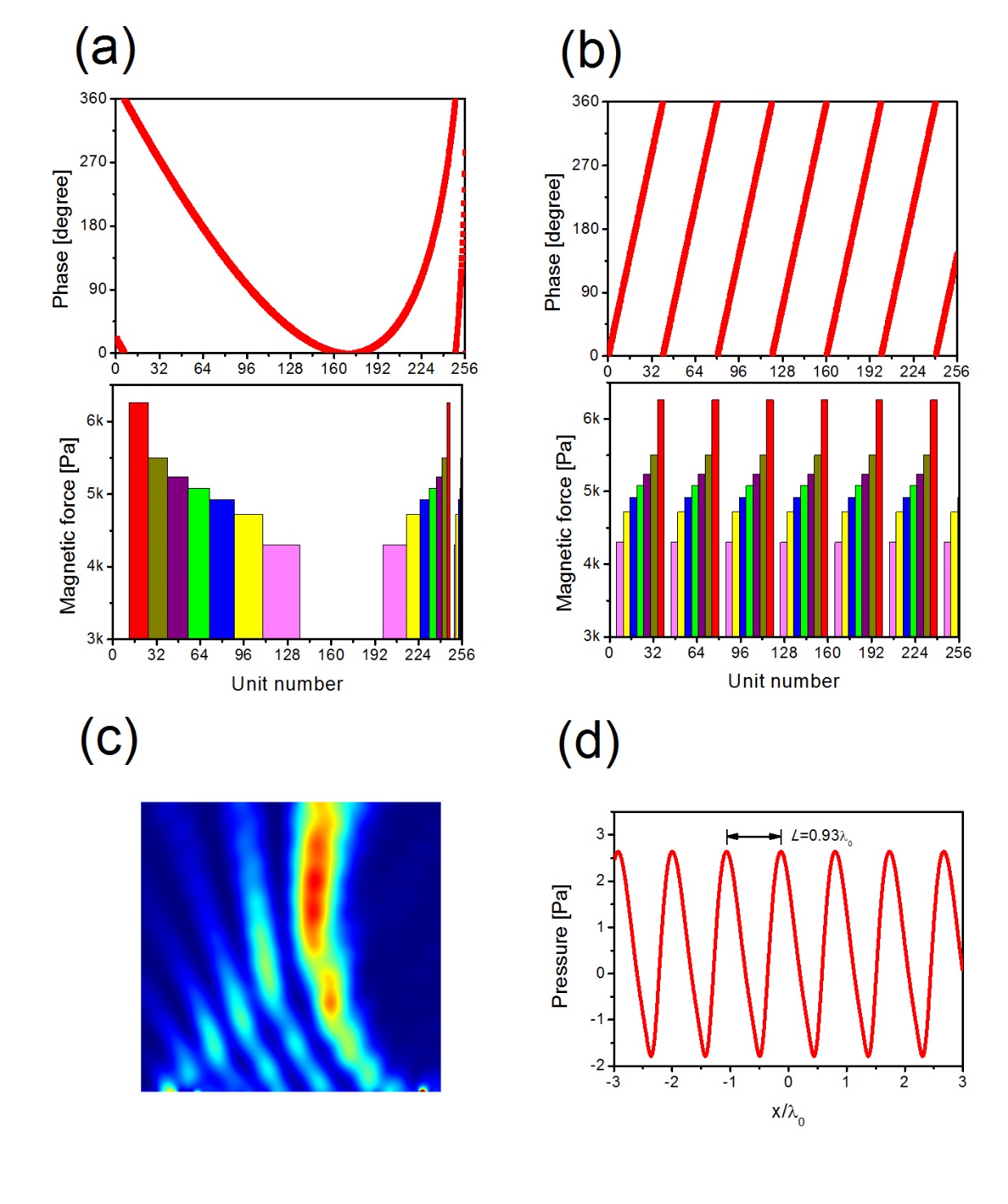
**

**Supplementary Figure 7 Versatile wavefront manipulation by the magnetic-control metasurface.** (a) The theoretical phase profiles and the applied magnetic forces are given for acoustic wave bending. (b) The theoretical phase profiles and the applied magnetic forces are given for generating the surface wave. (c) The pressure intensity distribution for bending beam in 400Hz. (d) The pressure distribution along y=100mm for propagation mode conversion in 400Hz.

**SUPPLEMENTARY NOTES**

**Supplementary Note 1. The design of unit with desired phase delay in broadband.** To steering the wavefront based on the concept of phase engineering, the basic requirement is to obtain arbitrary phase delay in the range of 2π regime. Practically, the continuous phase delay states are replaced by several discrete states. Here, we choose to design eight basic units with the phase increment of π/4. As mentioned in the manuscript, the reflected phase of the membrane-type acoustic metamaterials is closed related with the stress states. Thus, in help of tuning the out-of-plane magnetic force, the membrane’s tension will be changed and the reflected phase is readily tailored as shown in Supplementary Figure 1(a). For example, eight basic units, whose reflected phase in 400Hz are 0, π/4, π/2, 3π/4, π, 5π/4, 3π/2, and 7π/4, respectively, are selected from a vertical line in the black arrow. To further exhibit the reflected properties of the eight discrete units, we calculate the reflected acoustic wave field as shown in Supplementary Figure 1(b). The black auxiliary line indicates that the abrupt phase from the eight units follow linear variation as expected. Considering that the thickness of the units is much smaller than the wavelength (λ/85), the units’ vibro-acoustic coupling behaviors are separately shown in the bottom. It can be observed that the structural responses to the excitation are different, resulting in various abrupt phase. It is noted that the No. 5 unit is a critical state corresponding to the resonant frequency. When the driving frequency is close to the resonant frequency, the oscillation of the membrane experiences 180 degree phase transition and the movement of membrane structure’s movement. To expand the operative frequency, the larger magnetic force is applied for shifting the structural vibration properties. As shown in Supplementary Figure 2, the magnetic-control reflected wave phase is realized in 800Hz and 1600Hz. The red circles represent eight states, in which discrete phase shifts cover the full 2π span with steps of π/4. For each frequency component, the eight states is the foundation for wavefront steering.

**Supplementary Note 2. The demonstration of magnetic-control reflected angle in broadband.** In help of the new degree of freedom by the magnetic field, the proposed metasurface shows great flexibility in steering the wavefront. By introducing the abrupt phase along the metasurface, the reflected direction can be easily tuned. Inferred from the Eq. (6) in the manuscript, gradient phase is desired for anomalous reflection. To design a reflected angle of 42 degree in 400Hz, the theoretical phase profile is shown in the Supplementary Figure 3(a). Here, eight discrete states, mentioned above, are used to mimic the continuous phase variation. It is noted that the magnetic force distribution have obvious periodicity along the metasurface. When the same design target should be satisfied in other frequency, the period constant is elaborately adjusted by the magnetic field (Supplementary Figure 3(b) and (c)). For the same frequency component, magnetically tuned the phase gradient will result in reflection direction variation (Supplementary Figure 3(e) and (f)). To demonstrate the function of the magnetic-control metasurface, partial region (the dotted box) is plotted in the manuscript and the pressure along the y direction is given in Supplementary Figure 4. Based on the geometrical relationship, the reflected angle is calculated by: $\theta=arc\cos(\lambda/L)$. The *L* is the peak-to-peak distance along the y direction and λ  is the wavelength. Thus, the obtained reflected angles are 40 degree, 41.7 degree and 40.7 degree for 400Hz, 800Hz and 1600Hz, which is quite close to the design value 42 degree. Compared with the design reflected angles of 19 degree and 0 degree in 400Hz, the obtained reflected angles (20.8 degree and 0 degree) show good agreements.

**Supplementary Note 3. The demonstration of magnetic-control focal spot in broadband.** To achieve acoustic focusing by a planar structure, the arc-shaped phase profile is desired on the metasurface. Thus, the magnetic force distribution is switched from the periodic arrangement to the symmetrical arrangement. The location of the focal spot is determined by the radius of the equiphase surface. To keep the unchanged focal spot in broadband, the same equiphase surface is desired for each Fourier component of acoustic wave. However, acoustic wave passes through the same path with diverse phase difference for different frequencies. The low frequency acoustic wave need longer trajectory for the same phase delay. As shown in Supplementary Figure 5(a), (b) and (c), the dramatic changes of the reflected phase is required for high frequency. For fixed frequency, the shape of the equiphase surface is transformed for tunable focal spot (Supplementary Figure 5(e) and (f)). To verify the design strategy, the discrete magnetic forces are applied. To investigate the focusing effect, the pressure intensity (along x=0) is plotted in the Supplementary Figure 6 and the focal spot can be identified easily. The slight deviation between calculated focal length and theoretical value is due to the discrete phase profile. When the focal spot is far from the structure, the intensity of the focal spot is weaker. It can be understood that each unit of the metasurface is a secondary sound source, and the radiation intensity is inversely proportional to the distance. Besides, the energy is much more concentrated in higher frequency. This is due to that the error from phase discretization is smaller in high frequency.

**Supplementary Note 4. The implementation of the switchover functions by the magnetic field.** To guide sound energy along curved path *f(x)*, the bending acoustic beam is predicted by the caustic theory. A caustic is defined as an envelope to a family of tangents such that each point x at the plane y=0 can be functionally related to a point on the caustic via a tangent of slope θ. The desired trajectory is retrieved from tracing each individual caustic ray, and the phase gradient satisfies: . The theoretical phase distribution and the magnetic force distribution are plotted in the Supplementary Figure 7(a). The phenomena of the bending beam is observed by the pressure intensity pattern. Finally, the generation of the surface wave is explored. Surface wave can confined the energy along the interface. To meet the excitation condition, the surface should support the large transverse wave vector. Thus, the high phase gradient of π/20*p* is employed by the magnetic field. The theoretical phase distribution and the magnetic force distribution are plotted in the Supplementary Figure 7(b). To confirm the propagation mode conversion, the pressure distribution along y=100mm is plotted in 400Hz. The wave vector $k_{x}\text{=}1/L\approx1.08\lambda^{\text{-}1}$ is bigger than the wave vector $k_{0}$ in free space. It represents that the acoustic wave decays along the normal direction and is confined to propagate along the surface.

**Supplementary Movie 1.** The functionality of sound field scanning is achieved by the static magnetic field. By means of varying the periodicity of the magnetic-controlled phase, the reflected angle can be easily manipulated.

**Supplementary Movie 2.** The capability of focal spot control at will is demonstrated by changing the central position of the equiphase surface through the magnetic forces.
